# *SOCS1* Mutation Subtypes Predict Divergent Outcomes in Diffuse Large B-Cell Lymphoma (DLBCL) Patients

**DOI:** 10.18632/oncotarget.774

**Published:** 2012-12-09

**Authors:** Birgit Schif, Jochen K. Lennerz, Christian W. Kohler, Stefan Bentink, Markus Kreuz, Ingo Melzner, Olga Ritz, Lorenz Trümper, Markus Loeffler, Rainer Spang, Peter Möller

**Affiliations:** ^1^ Institute of Pathology, University of Ulm, Germany; ^2^ Institute of Functional Genomics, University Regensburg, Germany; ^3^ Institute for Medical Informatics, Statistics and Epidemiology, University of Leipzig, Germany; ^4^ Department of Hematology and Oncology, Georg-August-University Göttingen, Germany

**Keywords:** Lymphoma, DLBCL, *SOCS1* mutation

## Abstract

*Suppressor of cytokine signaling 1* (*SOCS1*) is frequently mutated in primary mediastinal and diffuse large B-cell lymphomas (DLBCL). Currently, the prognostic relevance of these mutations in DLBCL is unknown. To evaluate the value of the *SOCS1* mutation status as a prognostic biomarker in DLBCL patients, we performed full-length *SOCS1* sequencing in tumors of 154 comprehensively characterized DLBCL patients. We identified 90 *SOCS1* mutations in 16% of lymphomas. With respect to molecular consequences of mutations, we defined two distinct subtypes: those with truncating (*major*) and those with non-truncating mutations (*minor*), respectively. The *SOCS1* mutated subgroup or the *minor/major* subtypes cannot be predicted on clinical grounds; however, assignment of four established gene-expression profile-based classifiers revealed significant associations of *SOCS1 major* cases with *germinal center* and specific *pathway activation pattern* signatures. Above all, *SOCS1 major* cases have an excellent overall survival, even better than the GCB-like subgroup. *SOCS1 minor* cases had a dismal survival, even worse than the ABC gene signature group. The *SOCS1* mutation subsets retained prognostic significance in uni- and multivariate analyses. Together our data indicate that assessment of the *SOCS1* mutation status is a single gene prognostic biomarker in DLBCL.

## INTRODUCTION

Diffuse large B-cell lymphoma (DLBCL) is a heterogeneous group of rapidly growing neoplasms with an aggressive clinical course. An intensive multi-agent chemotherapy can cure ~40% of patients and the combination with anti-CD20 therapy has further improved outcome for additional 10-25% of patients [1-4]. Despite the marked progress in treatment response of DLBCL, 30% of patients remain with refractory, often incurable disease [2,5]. The variability in DLBCL outcome raises the question how one can identify these patient subsets. Currently, only few prognostic models that predict outcomes based on clinical data have been proposed [6,7]. Tissue-based classification schemes [8,9] take expression of distinct proteins into account to predict clinical course and treatment response in DLBCL patients; however, these do not adequately reflect the heterogeneity of DLBCL. To get a handle on the diversity of DLBCL several gene expression based classifiers have been devised [10-13]. The most widely used molecular subclassification [10] divides DLBCL into at least three prognostic relevant subtypes: one clinically aggressive subset that demonstrates a profile similar to activated peripheral blood B-cells (ABC), a second subtype with a better prognosis that is similar to normal germinal center B-cells (GCB), and a third subtype, referred to as PMBL [14] with an outcome that is superior to all other DLBCL subtypes [15,16]. While gene expression profiling can help to refine DLBCL classification into GCB, ABC, and PMBL subtypes with survival differences in patients [17], its use in clinical practice is not yet routine.

*Suppressor of cytokine signaling 1* (*SOCS1*) is frequently mutated in PMBL [18] and other lymphomas [19]. *SOCS1* inhibits janus kinase (JAK)/signal transducer and activator of transcription (STAT) signaling [20]. The C-terminal domain including the SOCS box is necessary for this function [21] and we have shown that mutations affecting this domain result in abnormal stabilization of JAK2 and dysregulation of JAK/STAT signaling [18]. Although the specific role in lymphomagenesis remains to be elucidated, *SOCS1* is a postulated tumor suppressor gene that is frequently inactivated by genomic mutations [22,20]. In case series and DLBCL cell lines, other groups have described *SOCS1* mutations [23-25]; however, the prognostic relevance of *SOCS1* mutations in DLBCL has not been addressed.

The aim of the present study was to assess the value of the *SOCS1* mutation status as a prognostic biomarker in a well-characterized cohort of DLBCL patients. We found that *SOCS1* mutation subtypes disclose significant differences in outcome. Thereby, assessment of the *SOCS1* mutation status represents a novel tumor-derived, single gene prognostic biomarker in DLBCL.

## RESULTS

**Figure 1:**
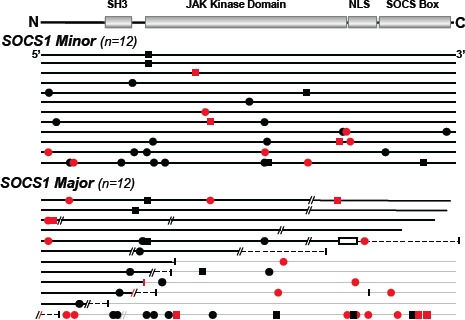
*SOCS1* Mutations in DLBCL *SOCS1* sequences of mutated DLBCL plotted and sorted by number and predicted severity of mutations (top row, encoded domains over coding regions). *SOCS1* coding region (length: 636bp) of each mutated samples is represented by a black line and symbols visualize the type and site of each mutation. Grey lines represent nonsense sequence after a mutation site, when appropriate. Circles are replacement substitutions, squares are silent mutations, diagonal lines are deletions, a box represents duplication, and vertical lines symbolize premature stop codons. Symbols are red when mutations occurred at consensus sites for somatic hypermutation. Note that *SOCS1* mutations are randomly distributed within the coding region. Abbreviations: SH3, Src homology 3; JAK, Janus kinase; NLS, nuclear localization signal; SOCS box, silencer of cytokine signaling box.

**Figure 2:**
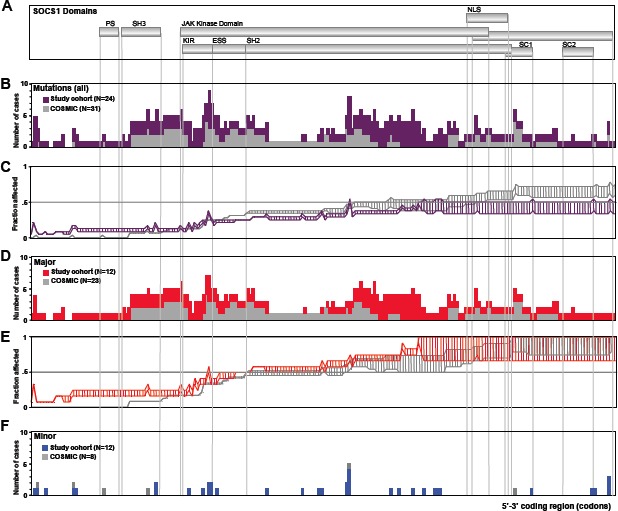
*SOCS1* Mutation Frequency A. *SOCS1* coding region depicted as scheme of functional domains. B. Frequency map compares regional distribution of *SOCS1* mutations in DLBCL cases from our cohort (purple bars, N=24) and all *SOCS1* mutated cases form the COSMIC database (grey bars, N=31); there are no mutational hotspots. While the KIR and SH2 regions within the JAK domain were frequently affected, mutations directly affecting the C-terminal SOCS box were rarely seen. C. Fraction of cases affected by downstream consequences of 5' mutations. The plot was constructed considering a range of predicted functional consequences of length-altering mutations (i.e., truncating and/or frameshift mutations) on downstream domains. The *bottom line* indicates a ‘conservative’ weighing where only the complete lack of C-terminally encoded domains is considered a deleterious event whereas in the *top line*, a more ‘aggressive’ weighing also accounted for alterations in domain position or partial disruptions of domains. Thus, the range between lines symbolizes the predicted spectrum of encoded downstream consequences of 5' mutations. Shown is a comparison of our study cohort (purple) and the COSMIC database (grey). Based on these graphs, cases with truncating or deleterious mutations that affected presence or position of C-terminal domains were considered *SOCS1 major* whereas those cases with non-truncating/non-foreshortening mutations (i.e., point mutations) were considered *SOCS1 minor*. D. Subgroup analysis of regional distribution of *SOCS1 major* mutations in our DLBCL cohort (red; N=12) in comparison to major cases in the COSMIC database (grey; N=23). Note the absence of mutational hotspots. E. Fraction of 5' C-terminal consequences in *SOCS1 major* subgroup in our cohort (red) and in the COSMIC database (grey). F. Fraction of mutations in the *SOCS1 minor* subgroups in our cohort (blue, N=12) and the COSMIC database (grey, N=3).

*SOCS1* mutations in DLBCL are frequent and do not cluster at mutational hotspots. Full-length sequencing of *SOCS1* revealed mutations in 24 (16%) of the 154 DLBCL samples (Table 1). Figure 1 summarizes the position and types of all *SOCS1* mutations within the coding region (detailed information in Supplemental Figure 1 and Supplemental Table 1). In total, we found 90 unique mutational events in these 24 individual cases (referred to as *SOCS1 mutant*). Five of 24 *SOCS1 mutant* cases carried a single mutation, whereas multiple mutations accumulated in the majority of cases. Mapping of the *SOCS1* mutations over the coding region and comparison with annotated mutations in the COSMIC database (Figure 2) demonstrated that mutations are spread throughout the coding region (Figure 2B). Although some domains were more frequently affected (Supplemental Table 2), there were no mutational hotspots. Specifically, *SOCS1* mutations in DLBCL mainly affected the region encoding the JAK domain and therein the *kinase inhibitory region* (KIR) and *Src homology 2* (SH2) subdomains. In addition, we noted that mutations are rarely located primarily in C-terminal domains; however, when consequences of (5') upstream mutations were taken into account (Figure 2C), the fraction of cases with predicted alterations in C-terminal domains increased substantially. The deleterious impact of truncations and/or frameshifts that alter longer stretches of the gene, affected in particular the SOCS box (range 35-50% of *SOCS1* mutant DLBCL). Thus, SOCS1 C-terminal domains are rarely affected by primary events; however, C-terminal domains are frequently mutated or lost due to more severe upstream mutations (detailed mutation frequencies are provided in Supplemental Table 2). Consequently, *SOCS1* sequence analysis implies different degrees of mutational severity, which can be visualized via the length of intact coding sequence (Figure 1). Accordingly, we defined *SOCS1 minor* as cases that harbor only non-foreshortening point mutations (12 of 24 *SOCS1* mutated cases in our cohort), and the *SOCS1 major* group as cases with truncating mutations or deletions that affect presence or position of C-terminal domains (12 of 24 *SOCS1* mutated cases in our cohort; Figure 2C). To account for these differences, we performed subgroup analyses based on the two mutation subtypes (*SOCS1 minor* vs. *major).*

**Table 1:** Demographic and Clinical Characteristics of the Study Cohort screened for *SOCS1* Mutations

Characteristic	Patient cohort n=154	%
**Age, years**		
Median (Range)	64.5 (3-93)	
<60 years	62	40.3
≥60 years	92	59.7
**Sex**		
Male	92	59.7
Female	62	40.3
**Ann Arbor stage**		
I and II	48	43.6
III and IV	62	56.4
**Lesions**		
EN only	20	20
N only	55	55
EN + N	25	25
**B symptoms**		
Absent	55	57.3
Present	41	42.7
IPI-score*		
0	12	10.5
1	49	43
2	31	27.2
>2	22	19.3
**Chemotherapy**		
ALL-like	13	11.5
CHOP-like	74	65.5
Other	15	13.3
None	11	9.7
**Radiotherapy**		
No	80	74.8
Yes	27	25.2
**Treatment response**		
CR+CRu	54	60.0
nC/SD	2	2.2
PR	15	16.7
PD	19	21.1
**Relapse**		
No	104	86.0
Yes	17	14.0

Abbreviations: ALL, acute lymphoblastic leukemia; CHOP, cyclophosphamide, doxorubicin, vincristine and prednisolone; CR, complete response; CRu, complete response unconfirmed; EN, extranodal; n, total number; N, nodal; nC/SD, no change/stable disease; PD, progressive disease; PR, partial response/resmission; IPI, international prognostic index. *for some patients not all IPI characteristics were available [i.e., a missing factor was set to ‘absent’ (0); therefore some patients with IPI 0/1 may have higher IPI-scores; see supplemental figure 2E]

*SOCS1* mutations preferentially occur at somatic hypermutation motifs. A previous study has postulated that *SOCS1* mutations might be the result of somatic hypermutation [25]. We checked the hotspot consensus motifs (RGYW/WRCY, DGYW/WRCH and WA/TW) known as somatic hypermutation target sites that result in single nucleotide substitutions (for details see methods and supplement). The numbers of point mutations per case ranged from 1-18 (Supplemental Table 3) and accumulated in the somatic hypermutation motifs (40,8 %). G/C nucleotides (n=64) were targeted more frequently compared to A/T (n=12) nucleotides (26 vs. 5 in somatic hypermutation motifs). In cases MPI-109 and MPI-153, one flanking region of each deletion was a somatic hypermutation site (Supplemental Figure 1, Supplemental Table 1). The frequency, location, and translational consequence of *SOCS1* mutations in our data sustain the hypothesis that these mutations are caused by aberrant somatic hypermutation.

*SOCS1* mutation status cannot be predicted on clinical grounds. Clinical characteristics of 154 DLBCL patients were analyzed with respect to their co-occurrence with *SOCS1* mutations. Table 2 lists the clinical and pathological features of *SOCS1 mutant* DLBCL patients. The immunophenotype of *SOCS1 mutant* DLBCL was characterized by presence of BCL2 (18/24, 75.0%) and BCL6 expression (23/23, 100.0% of evaluable cases). CD10 was expressed in 7 of 24 (29.2%), and MUM1 in 12 of 22 (54.5% of evaluable cases). Ki-67 immunostaining of *SOCS1 mutant* cases was variable but indicated a high proliferative rate. Seven of 23 cases (30.4%) displayed a BCL6 breakpoint by FISH analysis. Furthermore, comparison of *SOCS1* wild-type vs. *SOCS1* mutation subtypes did not allow discrimination based on any specific clinicopathological feature (Table 3). In consequence, the *SOCS1* status cannot be inferred from a basic panel of clinical parameters.

**Table 2:** Clinicopathological Characteristics of Cases with *SOCS1* Mutations

ID	Age, Sex	DLBCL Subtype*	Immunohistochemistry					FISH			Gene Expression Signatures			
			CD10	BCL2	BCL6	MUM1	Ki-67	MYC	BCL6	t(14;18)	COO	mBL	PAP	CC
MPI-135	77, F	ANA	−	+	+	+	99	−	−	−	ABC	non-mBL	PAP-3	BCR
MPI-202	72, M	NOS	−	−	+	+	80	−	+	−	GCB	non-mBL	PAP-1	HR
MPI-166	54, M	NOS	+	+	+	+	30	−	−	+	GCB	non-mBL	mind-L	BCR
MPI-247	70, M	CB	−	+	+	+	90	−	+	−	un.	non-mBL	PAP-1	BCR
MPI-030	59, M	CB	−	+	+	+	60	−	−	−	GCB	non-mBL	PAP-1	HR
MPI-165	86, F	NOS	−	−	+	−	95	nIG	+	−	GCB	Intermed	mind-L	BCR
MPI-199	66, M	CB	−	+	+	+	50	−	−	−	ABC	non-mBL	PAP-1	HR
MPI-063	73, F	NOS	−	+	+	+	NA	−	NA	−	un.	non-mBL	PAP-3	OxP
MPI-092	58, F	CB	−	+	+	+	65	−	−	−	GCB	non-mBL	mind-L	OxP
MPI-157	40, M	NOS	−	+	+	−	45	−	+	−	un.	non-mBL	PAP-2	NA
MPI-046	59, F	NOS	+	+	+	−	80	nIG	−	+	GCB	Intermed	PAP-3	BCR
MPI-134	29, F	CB	−	−	+	+	85	−	−	−	GCB	non-mBL	PAP-1	HR
MPI-105	60, F	CB	−	+	+	−	75	−	+	−	GCB	non-mBL	PAP-1	HR
MPI-241	40, F	NOS	−	+	+	−	80	−	+	−	GCB	non-mBL	mind-L	BCR
MPI-122	18, M	CB	−	+	+	+	90	−	−	−	GCB	non-mBL	PAP-1	HR
MPI-220	65, F	NOS	+	+	+	NA	90	IG	−	−	GCB	mBL	mind-L	NA
MPI-248	56, M	IB	+	+	+	−	50	nIG	−	+	GCB	Intermed	mind-L	BCR
MPI-136	32, M	CB	−	+	+	−	95	−	−	−	GCB	non-mBL	PAP-1	HR
MPI-137	18, M	NOS	−	+	+	+	85	−	−	−	GCB	non-mBL	PAP-1	NA
MPI-207	27, M	NOS	−	−	+	+	70	−	−	−	GCB	non-mBL	PAP-1	HR
MPI-036	70, F	CB	+	+	+	−	98	IG	−	+	GCB	Intermed	mind-L	BCR
MPI-153	78, F	CB	−	−	NA	NA	50	−	+	−	GCB	non-mBL	PAP-1	BCR
MPI-102	21, F	NOS	+	+	+	−	80	nIG	−	−	GCB	non-mBL	PAP-1	NA
MPI-109	42, M	CB	+	−	+	−	75	−	−	−	GCB	non-mBL	PAP-4	BCR

Abbreviations: ABC, activated B cell; ANA, anaplastic; CB, centroblastic; CC, consensus cluster signature; COO, cell-of-origin signature; DLBCL, diffuse large B-cell lymphoma; F, female; FISH, fluorescence in situ hybridisation; GCB, germinal center B cell; HR, host-response; IB, immunoblastic; ID, MPI-number from MMML cohort [11]; IG, immunoglobulin rearrangement of *MYC* gene; Intermed., intermediate signature; Ki-67, proliferation index in percent; M, male; mBL, molecular-Burkitt signature; mind-L, molecularly individual lymphoma; NA, not available; nIG, non-IG type of *MYC*-rearrangement; NOS, DLBCL not otherwise specified; OxPhos, oxidative-phosphorylative pathway; PAP, pathway activation pattern signature; t(14;18), *BCL2-IGH* translocation; un., unclassified; cut-offs for BCL2-, BCL6- and MUM1-negativity by immunohistochemistry were ≤25% and 0% for CD10-negativity. *according to Hummel et al [11].

**Table 3:** Clinical Characteristics of Patient Subsets based on *SOCS1* Status

	WT (n=130)		*SOCS1 Mut.* (n=24)		*SOCS1 Maj.* (n=12)		*SOCS1 Min.* (n=12)		*P*	*P*	*P*
Characteristic	No.	%	No.	%	No.	%	No.	%	WT vs. *Mut.*	WT vs. *Maj.*	WT vs. *Min.*
**Age, years**											
Median (Range)	66,0 (3-93)		58.5 (18-86)		56.5 (18-78)		62.5 (29-86)		0.46	0.13	0.66
<60	48	36.9	14	58.3	8	66.7	6	50	0.07	0.06	0.37
≥60	82	63.1	10	41.7	4	33.3	6	50			
**Sex**									0.37	0.54	0.54
Male	80	61.5	12	50	6	50	6	50			
Female	50	38.5	12	50	6	50	6	50			
**Ann Arbor stage**									1.0	1.0	1.0
I and II	41	44.1	7	41.2	4	44.4	3	37.5			
III and IV	52	55.9	10	58.8	5	55.6	5	62.5			
Lesions									0.73	0.91	0.75
EN only	16	18.9	4	26.7	2	25	2	28.6			
N only	48	56.5	7	46.6	4	50	3	42.9			
EN + N	21	24.7	4	26.7	2	25	2	28.6			
**B symptoms**									0.40	0.28	1
Absent	48	59.3	7	53.8	3	37.5	4	57.1			
Present	33	40.7	8	61.5	5	62.5	3	42.9			
**IPI-factors**									0.79	0.5	1
0,1	51	51.5	10	58.8	6	66.6	4	50			
≥2	46	46.5	7	41.2	3	33.3	4	50			
**Chemotherapy**									0.11	0.48	0.49
ALL-like	12	12.8	1	5.3	1	11.1	-	-			
CHOP-like	59	62.8	15	78.9	7	77.8	8	80			
Other	13	13.8	2	10.5	1	11.1	1	10			
None	10	10.6	1	5.3	-	-	1	10			
**Radiotherapy**									1	0.24	0.44
No	66	75	14	73.7	5	55.6	9	90			
Yes	22	25	5	26.3	4	44.4	1	10			
**Treatment response**									0.78	0.47	0.65
CR	36	47.4	7	50.0	6	66.7	1	20			
CR, unconfirmed	9	11.8	2	14.3	1	11.1	1	20			
nC/SD	2	2.6	-	-	-	-	-	-			
PR	13	17.1	2	14.3	1	11.1	1	20			
PD	16	21.1	3	21.4	1	11.1	2	40			
**Relapse**									0.74	1.0	1.0
No	86	85.1	18	90	9	90	9	90			
Yes	15	14.9	2	10	1	10	1	10			

Abbreviations: ALL, acute lymphoblastic leukemia; CHOP, cyclophosphamide, doxorubicin, vincristine and prednisolone; CR, complete response; EN, extranodal; IPI, international prognostic index; Maj., *major*; Min.; *minor*; Mut., *mutant*; n, total number; N, nodal; nC, no change; No., number of cases; PD, progressive disease; PR, partial response; SD, stable disease; *WT*, wild-type; *P* values refer to Fisher's exact test (including all characteristics except in chemotherapy: CHOP-like vs. non-CHOP and in treatment response CR/CR, unconfirmed vs. nC/SD/PR/PD).

*SOCS1 major* mutations exhibit the GCB gene expression signature. We correlated four established gene expression classifications (COO, mBL, CC, PAP) with the presence of *SOCS1* mutations (Table 4). Each expression-based signature captures a different aspect of DLBCL biology that may track with the *SOCS1* mutation status. The COO signature divided the 24 *SOCS1 mutant* cases into 19 (79.2%) GCB-like lymphomas and 5 (20.8%) non-GCB lymphomas. Strikingly, all 12 *SOCS1 major* cases show a GCB signature, whereas those with *SOCS1 minor* mutations showed no trend for a specific COO signature. *SOCS1* mutation status was at least in this DLBCL cohort not associated with the mBL signature (Table 4). Additionally, *SOCS1* mutations did not occur uniformly distributed over the PAP classification scheme (*P*=0.003; Fisher's exact test). Specifically, 12 *SOCS1 mutant* cases displayed the PAP-1 signature [n=^12^ (32%) of all 37 PAP-1 vs. n=^12^ (10%) of 117 non-PAP-1; *P*=0.003]. Further analysis by *SOCS1* mutation subtype revealed that 58.3% of the *SOCS1 major* cases display the PAP-1 pattern. There was no specific association with any CC signature (i.e. OxPhos, BCR, HR) in *SOCS1 mutant, SOCS1 major or SOCS1 minor* DLBCL cases. Together, examination of four established expression signatures revealed that *SOCS1 mutant* cases and in particular *SOCS1 major* cases accumulate in GCB and PAP-1 lymphomas. Notably, all *SOCS1 major* cases harbored the GCB signature.

**Table 4:** Gene-Expression Signatures of Patient Subsets based on *SOCS1* Status

	WT		*SOCS1 Mut.*		*SOCS1 Major*		*SOCS1 Minor*		*P*	*P*	*P*
Signatures	No.	%	No.	%	No.	%	No.	%	WT vs. *Mut.*	WT vs. *Maj.*	WT vs. *Min.*
**COO**									0.0006	<0.001	0.23
GCB	51	39.2	19	79.2	12	100	7	58.3			
ABC	47	36.2	2	8.3	-	-	2	16.7			
Uncl.	32	24.6	3	12.5	-	-	3	25			
**mBL**									1	0.6	1
mBL	9	6.9	1	4.2	1	8.3	-	-			
non-mBL	90	69.2	19	79.2	9	75	10	83.3			
Interm.	31	23.8	4	16.7	2	16.7	2	16.7			
**CC**									0.81	1	0.75
BCR	66	55.0	10	50.0	5	56	5	45.5			
HR	38	31.7	8	40.0	4	44	4	36.4			
OxPhos	16	13.3	2	10.0	-	-	2	18.2			
**PAP**									0.003	0.006	0.13
BL-PAP	10	7.7	-	-	-	-	-	-			
Mind-L	38	29.2	7	29.2	4	33.3	3	25			
PAP-1	25	19.2	12	50.0	7	58.3	5	41.7			
PAP-2	27	20.8	1	4.2	-	-	1	8.3			
PAP-3	21	16.2	3	12.5	-	-	3	25			
PAP-4	9	6.9	1	4.2	1	8.3	-	-			

Abbreviations: ABC, activated B cell; BCR, B cell receptor and activation; BL-PAP, Burkitt lymphoma-pathway activation pattern; CC, consensus cluster; COO, cell-of-origin signature; GCB, geminal center B-cell; Interm., intermediate; HR, host-response; mBL, molecular Burkitt lymphoma defined as 95% similar to Burkitt lymphoma [11]; Mind-L, molecularly individual lymphoma; Mut., *mutant*; No., number of cases; Uncl., unclassified; non-mBL, less than 5% similarity to Burkitt lymphoma [11]; OxPhos, oxidative-phosphorylative pathway; PAP, pathway activation pattern; *WT*, wild-type; *P* values refer to Fisher's exact test.

**Figure 3:**
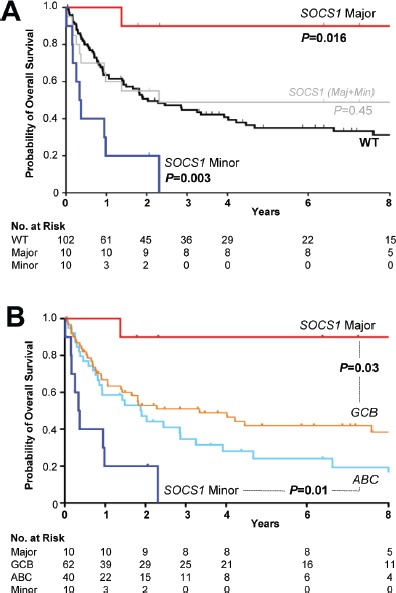
Kaplan-Meier Survival Estimates According to *SOCS1* Mutation Status A. The presence of *SOCS1* mutations (grey) in DLBCL patients was not associated with different overall survival compared to *SOCS1* wild-type (black; =0.45). Overall survival among patients with tumors harboring *SOCS1 major* mutations (red) was better when compared with *SOCS1* wild-type (*P*=0.016). DLBCL cases with *SOCS1 minor* mutations (blue) had a poor overall survival when compared to *SOCS1* wild-type cases (*P*=0.003). B. Relationship of overall survival according to *SOCS1* mutation type and cell-of-origin expression signature. Clinical outcome of *SOCS1 major* patients (red line) differed significantly from patients with a GCB signature (orange line; *P*=0.03). Clinical outcome of patients with *SOCS1 minor* patients (dark blue line) was significantly worse when compared to patients with an ABC signature (light blue line; *P*=0.01). All *P* values from Cox regression models.

**Figure 4:**
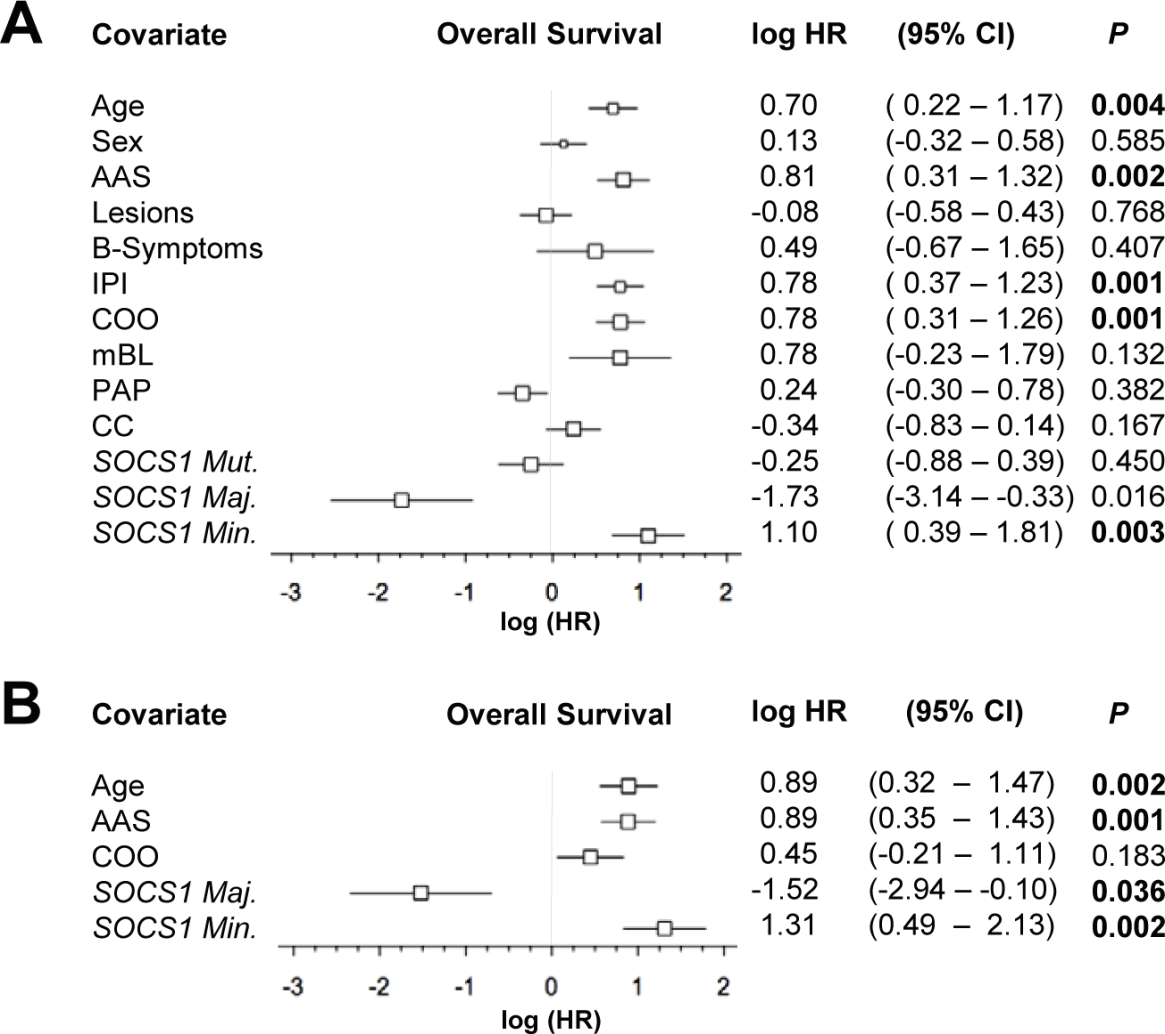
Forest plot of log hazard ratios (HR) for overall survival according to baseline clinical characteristics, assigned gene expression signatures and *SOCS1* gene status as well as mutation types A. Univariate and B. multivariate Cox proportional hazards regression models. Abbreviations: AAS, Ann Arbor Stage; IPI, international prognostic index; COO, cell-of-origin signature; mBL, molecular Burkitt signature; PAP, pathway activation pattern signature; CC, consensus cluster signature; CI, confidence interval. Maj., *major*; Min.; *minor*; Mut., *mutant*.

*SOCS1 major* and *minor* mutated cases differ in clinical outcome. At the time of data evaluation, clinical follow up information was available for 122 of 154 patients (~80% of the cohort). The median follow-up time of patients was 21.8 months (1.7 years; range: 0-22.3 years). Seventy-eight patients had died of disease (~51%) and 46 patients were either alive or lost to follow-up (censored). Based on combination chemotherapy carried out in the pre-rituximab era, the patients were not uniformly treated (Table 1) [11,26]. There was no difference in overall survival between *SOCS1 mutant* and *SOCS1* wild-type cases (Figure 3A, *P* = 0.45). Interestingly, the subgroups *SOCS1 major* and *SOCS1 minor* showed marked survival differences to *SOCS1* wild-type patients, however, in different directions: While overall survival was longer in patients with lymphomas that harbor *SOCS1 major* mutations, it was shorter in patients with *SOCS1 minor* mutations (Figure 3A). For quantitative comparison of prognostic effects, we determined the log hazard ratios using univariate Cox proportional hazard regression models (Figure 4A). In line with Figure 3, we observed that *SOCS1* gene mutation status by itself has little prognostic impact; however, *SOCS1* mutation subtypes herald different prognostic fates. More importantly, the comparison of hazard ratios across prognostic predictors showed that the *SOCS1* status is a strong factor. It is due to these strong effects that the *SOCS1* status reached statistical significance in spite of the relatively small number of cases in these two subgroups.

Patients with GCB-like lymphomas have a better prognosis than those with ABC-like lymphomas [10]. Notably, all our *SOCS1 major* cases were of the GCB-like type, suggesting that this might explain the survival difference. We plotted Kaplan-Meier curves for overall survival of *SOCS1 major* and *SOCS1 minor* against the *SOCS1* wild-type subgroup that we additionally separated into GCB- and ABC subgroups (Figure 3B). These curves suggest that *SOCS1 major* cases carry a good prognosis also when compared to GCB-*SOCS1* wild-type cases only, whereas *SOCS1 minor* cases have a poor prognosis even when compared to ABC-*SOCS1* wild-type cases. Moreover, the prognostic value of the COO signature remains intact when reduced to *SOCS1* wild-type cases suggesting that it cannot be attributed to the *SOCS1* mutation status alone. We corroborated this observation in a multivariate Cox model. With respect to outcome, the *SOCS1* mutation status had significant prognostic information independent of the covariates age, AAS, and COO signature (Figure 4B). Together these analyses demonstrated that *SOCS1 major* status is a predictor for better survival whereas *SOCS1 minor* status is a predictor for worse survival.

## DISCUSSION

Here, we evaluated the prognostic value and molecular characteristics of *SOCS1* mutations in a well-characterized cohort of 154 DLBCL patients. We show that *SOCS1* mutational subtypes are associated with divergent outcomes as well as distinct expression signatures. Thereby, *SOCS1* mutation status is a novel tumor-derived, single gene biomarker with molecular and prognostic implications in DLBCL.

Full-length *SOCS1* sequencing revealed 90 mutations in 24 of 154 DLBCL cases (16%), which is in line with *SOCS1* mutation frequencies in smaller DLBCL series (7/26 [25] and 8/33 [24]). Recently, two groups reported genome-wide mutation analyses of DLBCL with highly concordant results [23,27]. Remarkably, whole-exome sequencing disclosed *SOCS1* gene mutations in three DLBCL patients [23]. Here, we observed the accumulation of single nucleotide substitutions at sites preferentially targeted by somatic hypermutation. Given that frequencies and patterns are compatible with other hypermutated genes [28], our findings support the notion that germinal-center experienced lymphomas are prone to acquire *SOCS1* gene mutations by somatic hypermutation [25]. This conclusion has recently been confirmed using genome-wide approaches [29]. In addition, the spread of mutations over the coding region sustains the hypothesis that *SOCS1* is a tumor suppressor [20].

From a clinical perspective, our key finding is that the prognosis of patients with *SOCS1* mutations strongly depends on the nature of the mutation. We organized mutations by the lengths of intact encoded sequence (Figure 1, 2), thus splitting the cases in two groups: *SOCS1 major*, which has a good prognosis and *SOCS1 minor*, which has a poor prognosis (Figure 3, 4). While these findings are promising, several aspects should be taken into account: First, despite being the largest *SOCS1* mutation study in DLBCL to date, the number of cases in mutated subgroups is relatively small. While this is a typical side effect of highly resolved molecular stratifications, the estimated hazard ratios were so large that the survival difference reached statistical significance both in uni- and multivariate analyses (Figure 4). Second, determination whether a specific mutation was mono- or biallelic was precluded due to the presence of varying amounts of non-neoplastic cells in the tumor samples. This biologically imposed limitation should however not lessen the value of the biomarker and rather simplify the implementation process in terms of ease of diagnostic assessment. Third, we are not able to provide a specific biological explanation for the observed prognostic differences in the *SOCS1* mutation subgroups. It is tempting to hypothesize that the more severe, truncating mutations as seen in the *SOCS1 major* cases result in loss of its inhibitory function (i.e., resulting in prolonged activation of JAK/STAT signaling). For example, from *SOCS1 major* mutated cell lines we know that this is the case [18,22]. However, one has to acknowledge that the prognostic differences are not necessarily a linear consequence of the underlying molecular pathology and clearly not of the natural course of the disease. Rather, we observe a composite effect that includes at least chemotherapy-induced alterations as well as variability in response to treatment. To guide the design and interpretation of future functional studies, we abstain from speculations and merely point out that *SOCS1 major* vs. *SOCS1 minor* mutations are associated with increased vs. reduced overall survival, respectively. Fourth, the retrospective nature of this study does not account for treatment advances achieved by rituximab [3]. Interestingly, to date the majority of established prognostic factors in DLBCL remained prognostic – also in R-CHOP based studies [1]. Therefore, the strength of the observed associations between *SOCS1* mutation subtypes and overall survival makes *SOCS1* sequence analysis a promising prognostic biomarker candidate in DLBCL. Finally, the lack of mutational hotspots requires that full-length sequencing of *SOCS1* must be done to determine the *SOCS1* mutation status. Due to the shortness of this 636bp gene, however, this is easily feasible (also from DNA extracted from formalin fixed, paraffin embedded tumor tissue) and, therefore, should encourage independent validation in a larger and rituximab-treated cohort.

In summary, we have shown that *SOCS1* mutation subtypes in DLBCL track with distinct gene expression signatures and predict divergent outcomes. Thereby the *SOCS1* gene mutation status is a novel tumor-derived, single gene biomarker with prognostic relevance in DLBCL.

## MATERIAL AND METHODS

### Regulatory Approval and Study Population

Ethics committees of all participating institutions approved the protocols of the network project “Molecular Mechanisms in Malignant Lymphoma” (MMML; see Supplemental Appendix 1). Descriptions of the MMML project protocols, histopathological review and classification according to WHO guidelines [30] have been part of prior publications with a different focus [11,26,31,32]. In this study the focus is *SOCS1* mutation analysis in tumor samples from a consecutive cohort of well-characterized DLBCL patients. Table 1 lists the clinical characteristics of the cohort.

### PCR design and SOCS1 sequencing

The *SOCS1* gene is divided in two exon regions: Exon 1 (length:104 bp) contains the 5' UTR (untranslated region) and exon 2 (length:1766 bp) contains part of the 5' UTR, the translation initiation site (position 705), the stop codon (position 1340=c.636), and the 3' UTR. Primers were M13-tailed (biomers.net, Ulm, Germany) and designed to capture a 761 bp PCR fragment covering the complete open reading frame (636bp): exon2-forward primer 5'-CACCCCCGGACGCTATG and exon2-reverse primer 5'-CCACATGGTTCCAGGCAAGTA. After initial touchdown PCR, the amplification product was processed by agarose gel purification using the peqGOLD Gel Extraction Kit (peqlab, Erlangen, Germany). Sanger DNA sequencing employed the *BigDye Terminator* v3.1 Kit on a 3130 Genetic Analyzer (both ABI, Carlsbad, CA). Dye signals were translated by the *KB™ Base Caller Software* and visualized using the *Sequencing Analysis Software* v5.4 (both ABI).

### SOCS1 sequence and mutation analysis

Forward and reverse sequences were manually analyzed by blasting the obtained sequence against the human *SOCS1* sequence (ENST0000332029; *SOCS1*-001; www.ensembl.org, last accessioned Oct 1^th^, 2012). After annotation of the nucleotide alterations, sequence information was translated into protein sequence (ExPASy translate tool, www.expasy.org/translate/, last accessed Oct 1^th^ 2012). Alterations were mapped over the open reading frame as well as the known SOCS1 protein domains (Figure 1, Supplemental Figure 1) [18,20,33,34]. By plotting previously published mutated cases in a similar fashion (www.sanger.ac.uk/genetics/CGP/cosmic; last accessed Oct 1^th^, 2012; *SOCS1*: status Sept 13^th^, 2011), we compared the distribution of sequence alterations (Figure 2). Additionally, the DNA sequence of mutated *SOCS1* cases was used to analyze the targeting of the somatic hypermutation mechanism at specific hotspot motifs [28]. We used a DNA pattern search tool to identify somatic hypermutation hotspots (Gene Infinity LLC: http://www.geneinfinity.org/sms/sms_DNApatterns; last accessioned Oct 1^th^, 2012). Specifically, these preferred hotspots include RGYW/WRCY (G:C is the mutable position; R=purine, Y=pyrimidine, and W=A/T) [35], DGYW/WRCH (G:C is the mutable position; D=G/T/A; H=T/C/A) [36] and WA/TW (A:T is the mutable position) nucleotide pattern at both DNA strands [37]; see also supplement.

### Gene expression signatures

Prior characterization of cases employed in this study cohort included gene expression profiling on the Affymetrix U133A platform (Affymetrix, Santa Clara, CA, USA); data set GSE4475 is available at http://www.ncbi.nlm.nih.gov/geo/, last accessed Oct 1^th^, 2012. Briefly, four independently applied expression-based classifiers were attached to each case. These four classifiers were: the cell-of-origin signatures (COO) [10,38], the molecular Burkitt signatures (mBL) [11], the pathway activation pattern signatures (PAP) [12] and the consensus clustering signatures (CC) [13]. Nomenclature of the assigned signatures followed original publications: for the COO signature the subsets were GCB, ABC and unclassified [10,38]; for the mBL signature the subsets were mBL, non-mBL and intermediate [11]; for the pathway activation pattern signature [12] the subsets were BL-PAP, PAP-1, PAP-2, PAP-3, PAP-4, Mind-L, and for the CC signatures the subsets were oxidative phosphorylation (OxPhos), B-cell receptor/proliferation (BCR), host response (HR) [13]. When the original description of a classifier was based on another gene-expression platform than the one used herein (Affymetrix U133A), systematic differences were accounted for by applying appropriate adjustment algorithms [12,38].

Prior to the assignment of CC labels, study-specific effects (e.g., scanner generation, calibration, and platform) were adjusted in all models. Briefly, data from Monti et al. [13] were shifted and scaled to result in the same mean and variance as found across patients in the reference samples from Hummel et al. [11]. Subsequently, classification was performed by learning a linear 3-Class model with the nearest-shrunken centroid algorithm [39] on the data set of Monti et al. [13]. Finally this classifier was applied on the DLBCL samples from the MMML cohort, assigning the appropriate CC labels. Comparisons of each expression-based signature and subtypes therein were performed as uni- and multivariate analysis (see below).

### Clinical features

To ascertain the representative nature of this study cohort, we applied established clinical criteria with respect to treatment: chemotherapeutic classes (CHOP-like, ALL-like), radiotherapy (yes/no), treatment response (complete remission, partial remission, stable disease, progressive disease) and relapse (yes/no).

### Statistical Analysis

Statistical analysis consisted of Fisher's exact test (association of mutation status with nominal factors in contingency tables) and t test (comparison of age). Due to the retrospective nature of this cohort, treatment strategies were not uniform [11]. Therefore, the performance of established prognostic factors was assessed in the study cohort (see Supplemental Figure 2). The Kaplan-Meier method was used to estimate overall survival and uni- as well as multivariate cox proportional hazards regression models were used to analyze survival data. Given survival times, final life status (alive or dead) and one (univariate) or more (multivariate) covariates, the regression models produce a baseline survival curve and covariate coefficient estimates with their standard errors, 95% confidence intervals, and significance levels. The covariates included in these analyses were (parenthesis provide values set to 1): age (≥60), LDH (upper limit of normal, >ULN), ECOG (>1), Ann-Arbor stage (AAS; III/IV), extranodal involvement (EN; >1), B-symptoms (yes); morphology (immunoblastic, ib); COO signature (non-GCB); mBL signature (non-mBL); PAP signature (non-PAP1); CC signature (non-BCR); *SOCS1* status (mutation positive); *SOCS1 major* and *SOCS1 minor*. Moreover, the international prognostic index (IPI) score was tested (≥2) [40] and due to partial incompleteness of the basic data matrix for individual IPI characteristics in some patients, statistical testing was performed assuming the more pessimistic situation [i.e. a missing factor was set to “absent” (0); therefore some patients with IPI 0/1 may have higher IPI scores; Supplemental Figure 2E]. In univariate analyses, covariates were examined for their previously acknowledged prognostic impact, when applicable. In a second step, we combined factors demonstrating significance in univariate assessment in a multivariate analysis. Log hazard ratios are provided with the 95% confidence intervals (CI); significance was defined as *P*<0.05.

## Supplementary Figures and Tables


