# Supplementary material for: SOCS1 Mutation Subtypes Predict Divergent Outcomes in Diffuse Large B-Cell Lymphoma (DLBCL) Patients
Source: Oncotarget. 2012 Dec 9;4(1):35–47. doi: 10.18632/oncotarget.774 (PMC3702206; doi:10.18632/oncotarget.774)
Supplement: Supplementary file 1 [file oncotarget-04-035-s001.pdf]

Supplemental Appendix 1. Members of the network project of the Deutsche Krebshilfe "Molecular Mechanisms in Malignant Lymphomas" (alphabetical order)

Pathology group: Thomas F.E. Barth[1], Heinz-Wolfram Bernd[2], Sergio B. Cogliatti[3], Alfred C. Feller[2], Martin L. Hansmann[4], Michael Hummel[5], Wolfram Klapper[6], Peter Möller[1], Hans-Konrad Müller-Hermelink[7], Ilske Oschlies[6], German Ott[20], Andreas Rosenwald[7], Harald Stein[5], Monika Szczepanowski[6], Hans-Heinrich Wacker[6]
Genetics group: Thomas F.E. Barth[1], Petra Behrmann[8], Peter Daniel[9], Judith Dierlamm[8], Stefan Gesk[10], Eugenia Haralambieva[7], Lana Harder[10], Paul-Martin Holterhus[11], Ralf Küppers[12], Dieter Kube[13], Peter Lichter[14], Jose I. Martín-Subero[10], Peter Möller[1], Eva M. Murga-Peñas[8], German Ott[20], Claudia Philipp[12], Christiane Pott[15], Armin Pscherer[14], Julia Richter[10], Andreas Rosenwald[7], Itziar Salaverria[10],Carsten Schwaenen[16], Reiner Siebert[10], Heiko Trautmann[15], Martina Vockerodt[17], Swen Wessendorf[16]
Bioinformatics group: Stefan Bentink[18], Hilmar Berger[19], Christian W Kohler[18], Dirk Hasenclever[19], Markus Kreuz[19], Markus Loeffler[19], Maciej Rosolowski[19], Rainer Spang[18]
Project coordination: Benjamin Stürzenhofecker[13], Lorenz Trümper[13], Maren Wehner[13]
Steering committee: Markus Löffler[19], Reiner Siebert[10], Harald Stein[5], Lorenz Trümper[13]

Affiliations: [1]Institute of Pathology, University Hospital of Ulm, Germany, [2]Institute of Pathology, University Hospital Schleswig-Holstein Campus Lübeck, Germany, [3]Institute of Pathology, Kantonsspital St. Gallen, Switzerland, [4]Institute of Pathology, University Hospital of Frankfurt, Germany, [5]Institute of Pathology, Campus Benjamin Franklin, University Medical Center Charité Berlin, Germany, [6]Institute of Hematopathology, University Hospital Schleswig-Holstein Campus Kiel/ Christian-Albrechts University Kiel, Germany, [7]Institute of Pathology, University of Würzburg, Germany, [8]University Medical Center Hamburg-Eppendorf, Hamburg, Germany, [9]Department of Hematology, Oncology and Tumor Immunology, University Medical Center Charité Berlin, Germany, [10]Institute of Human Genetics, University Hospital Schleswig-Holstein Campus Kiel/Christian-Albrechts University Kiel, Germany, [11]Division of Pediatric Endocrinology and Diabetes, Department of Pediatrics, University Hospital Schleswig-Holstein Campus Kiel / Christian-Albrechts University Kiel, Germany, [12]Institute for Cell Biology (Tumor Research), University of Duisburg-Essen, Germany, [13]Department of Hematology and Oncology, Georg-August University of Göttingen, Germany, [14]German Cancer Research Center (DKFZ), Heidelberg, Germany, [15]Second Medical Department, University Hospital Schleswig-Holstein Campus Kiel/ Christian-Albrechts University Kiel, Germany, [16]Cytogenetic and Molecular Diagnostics, Internal Medicine III, University Hospital of Ulm, Germany, [17]Department of Pediatrics I, Georg-August University of Göttingen, Germany, [18]Institute of Functional Genomics, University of Regensburg, Germany, [19]Institute for Medical Informatics, Statistics and Epidemiology, University of Leipzig, Germany, [20]Institute of Clinical Pathology, Robert-Bosch Hospital, Stuttgart, Germany

Supplemental Results:

Mutational analysis. A total of 90 mutations were observed in 24 cases Supplemental Table 1 and Supplemental Figure 1. These mutations were composed of 12 deletions (5 resulted in frameshifts), one duplication, one translation initiation-site mutation and 76 point mutations (3 truncating-, 20 silent-, 52 missense mutations and 1 single-nucleotide polymorphism rs149311). The ratio of replacement to silent mutations (56/20) was 2.8. Single base substitution consisted of 36 transitions and 40 transversions and the transition to transversion ratio was 0.9 (expected, 0.5, if random bases were incorporated during substitutions) Supplemental Table 3. Notably, the ratio of G/C (n=64) to A/T (n=12) in these substitutions was shifted towards a predominance of G/C mutations. This overrepresentation suggests that mutations arise through somatic hypermutation (see below). As an additional approach the base pair substitutions were analyzed using the SIFT score (sorting intolerant from tolerant)[1] which predicts whether an amino acid substitution affects protein function (Supplemental Table 1).

Analysis of somatic hypermutation. In total, 40.8% (31/76) of all point mutations were found in somatic hypermutation hotspot motifs[2-4], whereas 13.1% of the sequenced *SOCS1* region includes such somatic hypermutation motifs. When the type of mutation is put in the context of hotspot motifs, 23 were replacement and 8 silent mutations (ratio 3.4). Analysis by somatic hypermutation motifs showed that 32.9% (25/76) of the somatic hypermutation mutations were present in RGYW motifs. The guanine (G) in the RGWY motif represents 34 residues of 636 nucleotides in the *SOCS1* coding region (5.3%). Moreover, 5.3% (4/76) targeted G of the DGYW and 8.0% (6/76) affected adenine (A) of the WA motif. The frequency of G within DGYW was 14 residues of *SOCS1* coding region (636 bp; 2.2%, on both strands) and A within WA hotspot is represented with 35 residues (5.5%; on both strands). In summary, the somatic hypermutation mutation pattern in the *SOCS1* gene in DLBCL samples was skewed towards G substitutions in RGYW hotspot.

Single nucleotide polymorphism (SNP). In this study cohort, the SNP rs149311 was seen in 32% of the 154 samples. This frequency is in accord with the DLBCL-specific frequency of rs149311 which is reported with ~38% (P=0.51; Fisher's)[5]. The specific allele frequency in this cohort was GG (67%), GA (28%) and AA (4%) whereas GG (62%), GA (34%) and AA (4%) is the DLBCL-specific allele frequency in the literature[5].

Differences were not significant (*P*=0.774; Fisher's) and we also excluded an association of the rs149311 status with the *SOCS1* mutation type (*P*=0.12; Fisher's).

***SOCS1* mutations in the COSMIC database.** The COSMIC database contained 67 individual *SOCS1* mutation entries. These were derived from 49 samples composed of 31 patient samples and 18 cell lines (6 entries/samples contained no positional information). Regional analysis of *SOCS1 mutant* cases in comparison with the DLBCL cohort is provided in Figure 2 as well as Supplemental Table 2. Almost all cases (91.7%) had mutations that affected the region encoding the JAK-kinase domain and therein the majority of mutations directly affected the region encoding the SH2 subdomain (83%). In contrast, mutations affecting the 3' regions, encoding C-terminal domains such as the SOCS box (25%) or the recently discovered nuclear localization signal (NLS, 12,5%) were relatively rare (Supplemental Table 2).

Supplemental Table 1. Overview of *SOCS1* Mutations

Case	DNA	AA	Mutation (SIFT)
**Minor**			
MPI-135	c.195G>A	p.R65R	silent (.57)
MPI-202	c.195G>A	p.R65R	silent (.57)
MPI-166	c.258G>C	p.V86	silent (.37)
MPI-247	c.136C>T	p.P46S	missense (1)
MPI-030	c.7G>C	p.A3P	missense (.08)
	c.402C>T	p.R134R	silent (.88)
MPI-165	c.314A>G	p.D105G	missense (.03)
MPI-199	c.296G>A	p.G99D	missense (0)
MPI-063	c.16C>G	p.Q6E	missense (.75)
	c.318C>T	p.S106S	silent (1)
	c.347G>A	p.S116N	missense (0)
MPI-092	c.440T>G	p.L147R	missense (0)
	c.442T>C	p.F148L	missense (.3)
	c.630G>C	p.Q210H	missense (.06)
MPI-157	c.197G>A	p.R66H	missense (.16)
	c.348C>A	p.S116R	missense (0)
	c.429C>T	p.S143S	silent (1)
	c.447G>C	p.E149D	missense (.57)
MPI-046	c.5 T>C	p.V2A	missense (0)
	c.174C>A	p.F58L	missense (.46)
	c.187G>C	p.D63H	missense (.02)
	c.346A>G	p.S116G	missense (0)
	c.544A>C	p.I182L	missense (.05)
MPI-134	c.47C>A	p.A16E	missense (.89)
	c.50C>G	p.A17G	missense (.32)
	c.137C>A	p.P46Q	missense (.69)
	c.197G>A	p.R66H	missense (.16)
	c.202A>C	p.T68P	missense (.11)
	c.344T>C	p.L115P	missense (0)
	c.348C>T	p.S116S	silent (1)
	c.416G>A	p.G139D	missense (.15)
	c.600C>T	p.L200L	silent (1)

Case	DNA	AA	Mutation (SIFT)
**Major**			
MPI-105	c.46G>T	p.A16S	missense (.81)
	c.195G>A	p.R65	silent (.57)
	c.318C>G	p.S106R	missense (0)
	c.421_426del	p.R141_E142del	deletion
	c.484C>T	p.L162L	silent (1)
MPI-241	c.174C>T	p.F58F	silent (1)
	c.403_423del	p.F135_Y141del	deletion
MPI-122	c.4G>C	p.V2L	missense (.01)
	c.6A>G	p.V2	silent (1)
	c.29_37del	p.D10_A12del	deletion
	c.223_254del	p.D75_S85del	deletion
MPI-220	c.322_423del	p.R107_S140del	deletion
MPI-248	c.5T>C	p.V2A	missense (0)
	c.188A>C	p.D63A	missense (.01)
	c.195G>C	p.R65	silent (.57)
	c.347G>A	p.S116N	missense (0)
	c.403_405del	p.F135del	deletion
	c.362_420dup	p.?	duplication
	c.428 G>T	p.S143I	missense (.13)
MPI-136	c.178_180del	p.S60del	deletion
	c.184G>A	p.A62T	missense (0,56)
	c.354_643del	p.K118fs*38	deletion
MPI-137	c.243G>A	p.W81*	premature stop
	c.374G>T	p.S125I	missense (0)
MPI-207	c.164T>G	p. F55C	missense (.18)
	c.203_218del	p.T68fs*11	deletion
	c.300C>G	p.T100	silent (1)
	c.358G>T	p.A120S	missense (.75)
MPI-036	c.192C>G	p.Y64*	premature stop
	c.237C>G	p.F79L	missense (.01)
	c.416G>C	p.G139A	missense (.57)
MPI-153	c.127C>T	p.P43S	missense (.65)
	c.177_204del	p.S60fs*16	deletion
	c.374G>A	p.S125N	missense (.52)
	c.462C>A	p.Y154*	nonsense
	c.484C>A	p.L162M	missense (0)
MPI-102	c.49G>A	p.A17T	missense (.34)
	c.53_212del	p.A18fs*16	deletion
MPI-109	c.-6_15del	p.0?	deletion
	c.26C>G	p.A9G	missense (.87)
	c.35C>T	p.A12V	missense (.11)
	c.100G>T	p.A34S	missense (.79)
	c.107C>G	p.P36R	missense (.52)
	c.108_174del	p.A37fs	deletion
	c.181C>A	p.H61N	missense (.36)
	c.220C>G	p.L74V	missense (.16)
	c.256G>A	p.V86M	missense (.14)
	c.258G>C	p.V86	silent (.37)
	c.330C>G	p.N110K	missense (.47)
	c.387C>T	p.H129	silent (.23)
	c.447G>C	p.E149D	missense (.57)
	c.450G>A	p.L150L	silent (1)
	c.451C>G	p.L151V	missense (.56)
	c.456G>C	p.E152D	missense (.08)
	c.528G>C	p.E176D	missense (0)
	c.570C>T	p.N190	silent (.69)
	c.571C>T	p.L191	silent (.32)
	c.591C>T	p.N197	silent (.18)

Nomenclature follows Human Genome Variation Society (HGVS, http://www.hgvs.org/mutnomen/; last accessioned Oct 1th, 2012) and positional information refers to NM_003745 and ENSP00000329418 for DNA and amino acid, respectively. The SIFT (sorting intolerant from tolerant) score predicts whether an amino acid substitution affects protein function[1]. **Abbreviations:** AA, amino acid; c., affected position coding DNA; p., AA position; >, single base substitutions; _ range of changed sequence; del, deletion; dup, duplication; *, stop codon; fs, frame shift; red indicates mutations at somatic hypermutation motifs.

Supplemental Table 2. *SOCS1* Mutation Frequency by Protein Domains

				COSMIC cohort			DLBCL cohort			*SOCS1 Major*			*SOCS1 Minor*		
SOCS1 **Domain**	**AA**	**Interaction/ Binding site for**	**Ref**	**n=31 N**	**%**	**[range %]**	**n=24 N**	**%**	**[range %]**	**n=12 N**	**%**	**[range %]**	**n=12 N**	**%**	**[range %]**
Poly-Serine	26-32	O-glycosylation site	6	1	3.2	[3.2-3.2]	1	4.2	[8.3-12.5]	1	8.3	[16.7-25.0]	0	0.0	[0.0]
**SH3 domain**	34-47	Grb2		3	9.7	[9.7-9.7]	5	20.8	[20.8-25.0]	3	25.0	[25.0-33.3]	2	16.7	[0.0]
PRR (type I)	34-39	Grb2		2	6.5	[6.5-6.5]	2	8.3	[8.3-12.5]	2	16.7	[16.7-25.0]	0	0.0	[0.0]
PRR (type II)	41-47	Grb2		3	9.7	[9.7-9.7]	5	20.8	[20.8-25.0]	3	25.0	[25.0-33.3]	2	16.7	[0.0]
**JAK domain**	56-166	inhibition of kinase activity		22	71.0	[77.4-77.4]	22	91.7	[95.8-95.8]	12	100.0	[100.0-100.0]	11	91.7	[0.0]
KIR	55-66	High affinity binding to JAKs.	7	8	25.8	[29.0-29.0]	13	54.2	[58.3-62.5]	9	75.0	[75.0-83.3]	5	41.7	[0.0]
ESS	67-78	Required for pY1007 binding of JAKs		6	19.4	[32.3-32.3]	6	25.0	[29.2-33.3]	5	41.7	[50.0-58.3]	1	8.3	[0.0]
SH2	79-174	Required for pY1007 binding of JAKs		17	54.8	[77.4-77.4]	20	83.3	[87.5-87.5]	11	91.7	[100.0-100.0]	9	75.0	[0.0]
TEC-kinase	82	inhibition of kinase activity	8	3	9.7	[35.5-35.5]	1	4.2	[29.2-33.3]	1	8.3	[58.3-66.7]	0	0.0	[0.0]
Arginin	104	phophotyrosine binding site	9	2	6.5	[35.5-35.5]	0	0.0	[25.0-33.3]	0	0.0	[50.0-66.7]	0	0.0	[0.0]
**NLS**	159-173	nuclear localisation	10	4	12.9	[54.8-67.7]	3	12.5	[37.5-50.0]	3	25.0	[75.0-100.0]	0	0.0	[0.0]
**SOCS box**	161-210	association with Elongin B/C targets for proteasomal degradation:	11	9	29.0	[71.0-83.9]	6	25.0	[50.0-62.5]	4	33.3	[66.7-100.0]	3	25.0	[0.0]
SC-motif 1	174-182	Elongin B/C box	12	4	12.9	[61.3-77.4]	3	12.5	[37.5-54.2]	2	16.7	[66.7-100.0]	1	8.3	[0.0]
SC-motif 2	194-204	protection of SOCS1 from degradation		0	0.0	[58.1-74.2]	3	12.5	[37.5-54.2]	2	16.7	[66.7-100.0]	1	8.3	[0.0]

Abbreviations: AA, amino acids; Ref, supplemental reference; n, number of *SOCS1*-mutated cases; N, number of mutations in the indicated domain; COSMIC, Catalogue of Somatic Mutations in Cancer; DLBCL, diffuse large B-cell lymphoma; SH3, Src Homology 3 (XPpXP); PRR, proline rich-repeats contain diproline motifs PxxPxR (type I) and RPxPXXP (type II) and represent the defining determinants of the SH3 domain; JAK, Janus-kinase; KIR, kinase inhibitory region; ESS, extended SH2 subdomain; SH2, Src Homology 2; TEC, tyrosine kinase expressed in hepatocellular carcinoma; NLS, nuclear localization signal; SC, STAT-induced STAT inhibitor COOH-terminal; pY1007, phosphorylated tyrosine at position 1007. **Symbols:** % (n/N) percent of all mutations; [range %] percent of cases (per domain) with mutations that are predicted to encode for a C-terminally foreshortened SOCS1 protein. The range takes the spectrum of 5' mutational consequences into account (details see main paper). Briefly, the left number indicates a 'conservative' weighing where only the complete lack of C-terminally encoded domains is considered a deleterious event whereas the right number is derived from a more 'aggressive' weighing which also accounts for alterations in domain position or partial disruptions of domains. Here, ranges are provided by domain and a plot over the entire coding region is provided in Figure 2C of the main paper.

Supplemental Table 3. Overview of Transition to Transversion Ratio

Case	TS/TV all	TS/TV SHM	*P*
**Minor**			
MPI-135	1/0	0/0	
MPI-202	1/0	0/0	
MPI-166	0/1	0/1	
MPI-247	1/0	0/0	
MPI-030	1/1	0/0	
MPI-165	1/0	0/0	
MPI-199	1/0	1/0	
MPI-063	2/1	1/0	
MPI-092	1/2	1/0	
MPI-157	2/2	1/1	
MPI-046	2/3	2/0	
MPI-134	5/4	1/1	
**sum**	18/14	7/3	*0.4901*
**Major**			
MPI-105	2/2	1/2	
MPI-241	1/0	0/0	
MPI-122	1/1	1/1	
MPI-220	0/0	0/0	
MPI-248	2/3	1/1	
MPI-136	1/0	0/0	
MPI-137	1/1	0/1	
MPI-207	0/3	0/0	
MPI-036	0/3	0/2	
MPI-153	2/2	1/1	
MPI-102	1/0	0/0	
MPI-109	7/11	3/6	
sum	18/26	7/14	*0.5977*
**Total**	36/40	14/17	*1.0000*

Case-based distributions of transition to transversion ratios were compared using the Fisher's exact test.
Abbreviations: TS, transition; TV, transversion; SHM, somatic hypermutation

Supplemental References

1. Ng PC, Henikoff S. SIFT: Predicting amino acid changes that affect protein function. Nucleic Acids Res. 2003;31:3812-3814.

2. Rogozin IB, Kolchanov NA. Somatic hypermutagenesis in immunoglobulin genes. II. Influence of neighbouring base sequences on mutagenesis. Biochim.Biophys.Acta 1992;1171:11-18.

3. Rogozin IB, Pavlov YI, Bebenek K, Matsuda T, Kunkel TA. Somatic mutation hotspots correlate with DNA polymerase eta error spectrum. Nat.Immunol. 2001;2:530-536.

4. Rogozin IB, Diaz M. Cutting edge: DGYW/WRCH is a better predictor of mutability at G:C bases in Ig hypermutation than the widely accepted RGYW/WRCY motif and probably reflects a two-step activation-induced cytidine deaminase-triggered process. J.Immunol. 2004;172:3382-3384.

5. Butterbach K, Beckmann L, de Sanjosé S et al. Association of JAK-STAT pathway related genes with lymphoma risk: results of a European case-control study (EpiLymph). Br.J.Haematol. 2011;153:318-333.

6. De Sepulveda P, Okkenhaug K, Rose JL et al. Socs1 binds to multiple signalling proteins and suppresses steel factor-dependent proliferation. EMBO J. 1999;18:904-915.

7. Yasukawa H, Misawa H, Sakamoto H et al. The JAK-binding protein JAB inhibits Janus tyrosine kinase activity through binding in the activation loop. EMBO J. 1999;18:1309-1320.

8. Ohya K, Kajigaya S, Yamashita Y et al. SOCS-1/JAB/SSI-1 can bind to and suppress Tec protein-tyrosine kinase. J.Biol.Chem. 1997;272:27178-27182.

9. Giordanetto F, Kroemer RT. A three-dimensional model of Suppressor Of Cytokine Signalling 1 (SOCS-1). Protein Eng 2003;16:115-124.

10. Baetz A, Koelsche C, Strebovsky J, Heeg K, Dalpke AH. Identification of a nuclear localization signal in suppressor of cytokine signaling 1. FASEB J. 2008;22:4296-4305.

11. Hilton DJ, Richardson RT, Alexander WS et al. Twenty proteins containing a C-terminal SOCS box form five structural classes. Proc.Natl.Acad.Sci.U.S.A 1998;95:114-119.

12. Narazaki M, Fujimoto M, Matsumoto T et al. Three distinct domains of SSI-1/SOCS-1/JAB protein are required for its suppression of interleukin 6 signaling. Proc Natl Acad Sci U S A. 1998; 95:13130-4.

13. Rabinovich SG. Measurement errors and uncertainties : theory and practice. 2nd Springer-Verlag : AIP Press, New York, NY 2000:

Supplemental Figure 1. Overview of *SOCS1* Mutations.

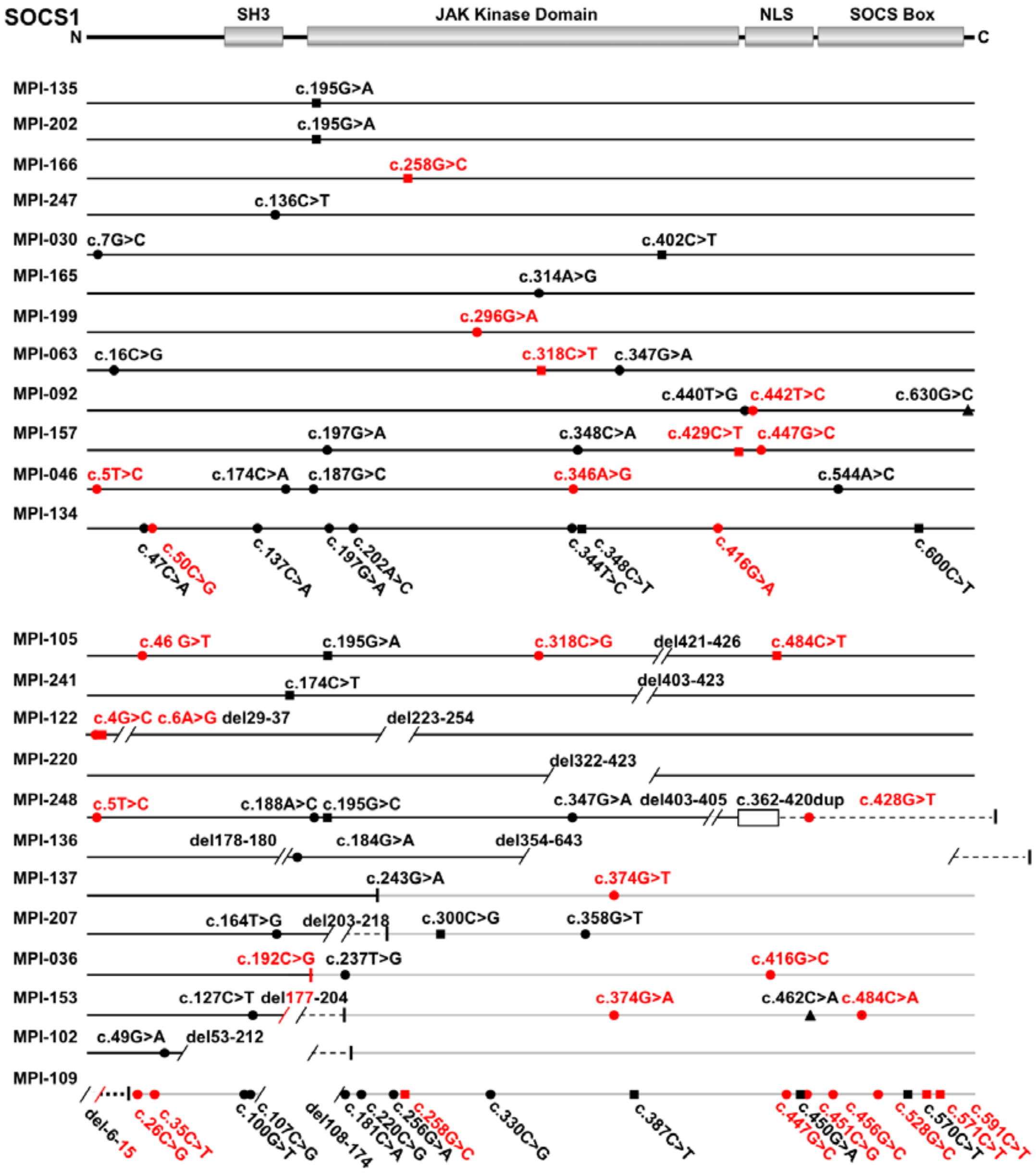

SOCS1 domains (top, schematic) and the *SOCS1* mutations per case (bottom). Each line represents the coding region of a *SOCS1* mutated case. DNA sequence mutation symbols are: circles (replacement mutations), squares (silent mutations), diagonal lines (deletion), box (duplication) or vertical lines (premature stop codon); grey lines represent non-sense sequence after a mutation, if appropriate. Red color (symbols and mutation) highlights mutations at somatic hypermutation motifs. Further annotations are c. (coding region), del (deletion) and > (nucleotide replacement to).

Supplemental Figure 2. Overall Survival in DLBCL Patients in the Study Cohort.

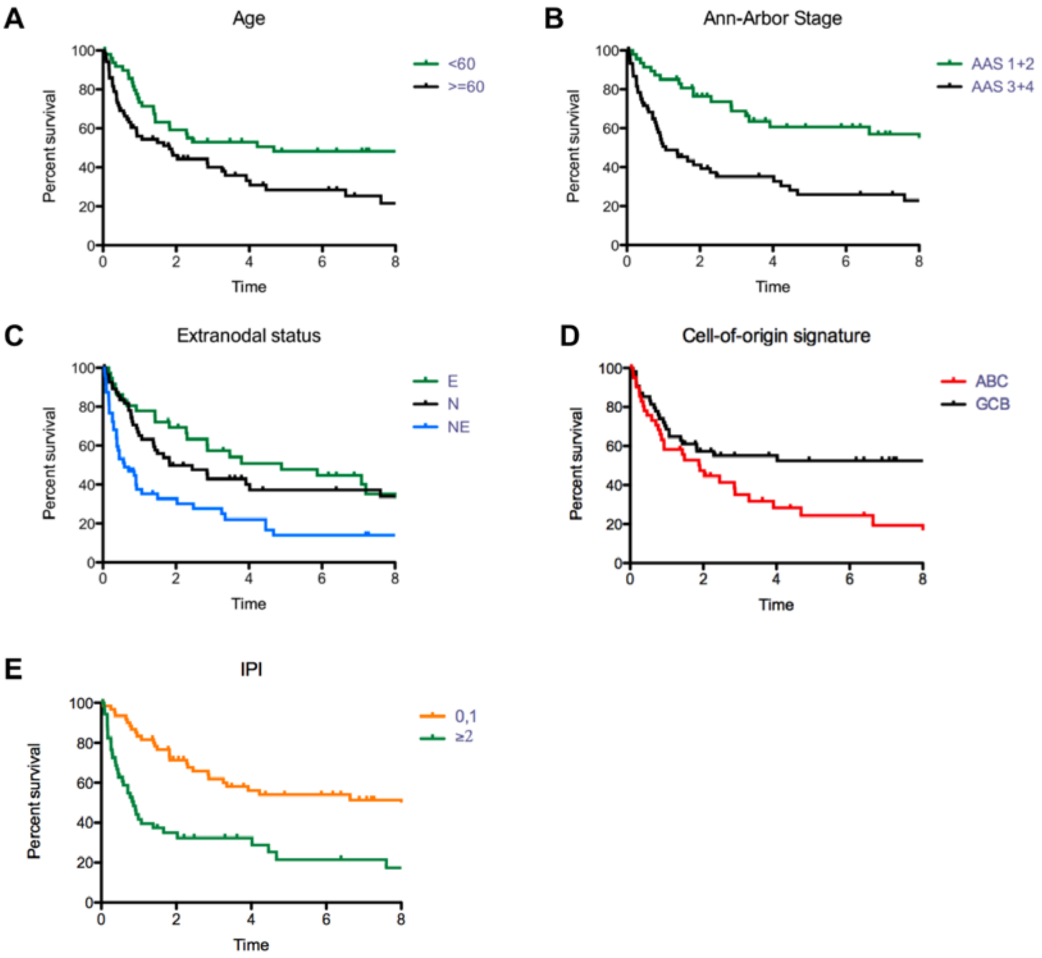

Comparison of overall survival is provided for the following parameters: Age in years (**A**; *P*=0.004), Ann-Arbor stage (AAS, **B**; *P*=0.0014), extranodal status (**C**; *P*=0.0078), cell-of-origin signature (**D**; *P*=0.005) and international prognostic index (IPI) (**E**; P<0.0001); time in years. Note: due to incompleteness of the basic data matrix for IPI characteristics, statistical testing was performed assuming the more pessimistic situation[13] [i.e. a missing factor was set to “absent” (0); therefore some patients with IPI 0/1 may have higher IPI scores].

Abbreviations: ABC, activated B-cell; E, extranodal; GCB, germinal center B-cell; N, nodal.
